# Gene Modules Co-regulated with Biosynthetic Gene Clusters for Allelopathy between Rice and Barnyardgrass

**DOI:** 10.3390/ijms20163846

**Published:** 2019-08-07

**Authors:** Most. Humaira Sultana, Fangjie Liu, Md. Alamin, Lingfeng Mao, Lei Jia, Hongyu Chen, Dongya Wu, Yingying Wang, Fei Fu, Sanling Wu, Weidi Wang, Chuyu Ye, Qian-Hao Zhu, Jie Qiu, Longjiang Fan

**Affiliations:** 1 Institute of Crop Science & Institute of Bioinformatics, College of Agriculture and Biotechnology, Zhejiang University, Hangzhou 310058, China; 2 Analysis Center of Agrobiology and Environmental Sciences, Faculty of Agriculture, Life and Environmental Sciences, Zhejiang University, Hangzhou 310058, China; 3 CSIRO Agriculture and Food, GPO Box 1700, Canberra, ACT 2601, Australia

**Keywords:** allelopathy, rice, barnyardgrass, biosynthetic gene cluster, WGCNA

## Abstract

Allelopathy is a central process in crop–weed interactions and is mediated by the release of allelochemicals that result in adverse growth effects on one or the other plant in the interaction. The genomic mechanism for the biosynthesis of many critical allelochemicals is unknown but may involve the clustering of non-homologous biosynthetic genes involved in their formation and regulatory gene modules involved in controlling the coordinated expression within these gene clusters. In this study, we used the transcriptomes from mono- or co-cultured rice and barnyardgrass to investigate the nature of the gene clusters and their regulatory gene modules involved in the allelopathic interactions of these two plants. In addition to the already known biosynthetic gene clusters in barnyardgrass we identified three potential new clusters including one for quercetin biosynthesis and potentially involved in allelopathic interaction with rice. Based on the construction of gene networks, we identified one gene regulatory module containing hub transcription factors, significantly positively co-regulated with both the momilactone A and phytocassane clusters in rice. In barnyardgrass, gene modules and hub genes co-expressed with the gene clusters responsible for 2,4-dihydroxy-7-methoxy-1,4-benzoxazin-3-one (DIMBOA) biosynthesis were also identified. In addition, we found three genes in barnyardgrass encoding indole-3-glycerolphosphate synthase that regulate the expression of the DIMBOA cluster. Our findings offer new insights into the regulatory mechanisms of biosynthetic gene clusters involved in allelopathic interactions between rice and barnyardgrass, and have potential implications in controlling weeds for crop protection.

## 1. Introduction

Allelopathy is a common biological phenomenon by which one plant influences the growth of neighboring plants through the release of allelochemicals into the rhizosphere [1]. In recent years, increasing attention has been paid to crop–weed allelopathy due to its promising application in weed management and crop protection [2,3]. Critical genetic mechanisms or pathways involved in crop- weed allelopathic interactions have been revealed [3,4]. One of these is the discovery of ‘operon- like’ biosynthetic gene clusters (BGCs) that are responsible for the coordinate formation of various allelochemicals [5] mediating allelopathic interactions [4,6]. It has been suggested that clustering of genes of the same biosynthetic pathway could help coordinate the expression of the component genes and minimize self-toxicity caused by accumulation of intermediate compounds. In addition, co-inheritance of whole pathway facilitates co-regulation of the genes involved, enables faster response to local environmental changes and finally enhances plant survival [5,7].

The unique features of known plant BGCs provide clues for identification of new gene clusters. For example, BGCs usually consist of two or more non-homologous genes that locate in an adjacent genomic region and encode different enzymes of the same pathway [5]. The functionally characterized gene clusters to date generally include 3–10 genes [8]. Another important characteristic of all the clusters identified so far is the presence of the P-450 monooxygenases (P450s) [5]. In addition, genes in the core cluster are coordinately regulated at the transcriptional level, and a co- expression pattern of the genes involved has been proved to be one of the critical criteria for successful gene cluster discovery [9]. Taking advantages of the above characteristics, state-of-the-art approaches, such as PlantiSMASH [10] and PlantClusterFinder [11], have been developed and successfully used in the identification of candidate gene clusters in plants.

As a staple food in the world, rice production is threatened by agricultural weeds, among which the *Echinochloa* complex is reported to be among the most persistent weeds associated with rice cultivation globally [12]. The allelopathic potential of rice (*Oryza sativa*) against weeds has been previously investigated [13] and several secondary metabolites, including fatty acids, indoles, momilactones, phenolics, and terpenes, have been demonstrated to be major allelochemicals in rice capable of suppressing the growth of barnyardgrass (*Echinochloa crus-galli*) [2]. Notably, among diterpenoid phytoalexins in rice, momilactone B has a much higher growth-inhibiting activity against surrounding plants than momilactone A, while phytocassanes showed no allelopathic effects, and are regarded as phytoalexins against pathogens in soils [14,15,16]. On the other hand, barnyardgrass also releases growth inhibitors that are harmful for rice growth [17,18]. Interestingly, both rice and barnyardgrass have evolved gene clusters that can biosynthesize allelochemicals against each other [4,6]. In the rice genome, genes encoding diterpene synthases and chemical-alteration enzymes, including P450s, formed the momilactone gene cluster [19]. The momilactone chemicals are significantly induced and inhibit the growth of barnyardgrass [20]. Meanwhile, 2,4-dihydroxy-7-methoxy-1,4-benzoxazin-3-one (DIMBOA) released from barnyardgrass has been confirmed to be a critical allelochemical against rice. Three copies of the DIMBOA clusters have been found in the barnyardgrass genome, and the genes within the cluster are significantly over-expressed in barnyardgrass co-cultured with rice. As many more new candidate gene clusters in plants have been predicted by the new approaches [10,11], it is of interest to know whether there are more BGCs involved in allelopathy between rice and barnyardgrass.

Following the discovery of BGCs an open question remained as to whether which other genes and regulatory factors are important in coregulating gene expression in these clusters and thus play roles in the modulating allelopathic interactions. For example, Yamamura et al. [21] found a bHLH transcription factor (*LOC_Os01g09990*) named as *DPF* in rice that acts as a master regulator of the biosynthesis of diterpenoid phytoalexin. Over-expression and knockdown of this transcription factor increased and decreased, respectively, the expression level of genes in both BGCs (momilactone, phytocassane), and consequently affected the accumulation of diterpenoid phytoalexins. Analysis of co-expression gene network has been widely used to study the relationships between functional modules and plant biological processes, and to uncover gene regulatory mechanisms in both model and non-model plants [22]. As genes involved in specialized metabolic pathways are often coordinately regulated, co-expression has been adopted to discover genes in plant-specialized metabolism [9]. Notably, WGCNA (weighted gene co-expression network analysis) is an efficient co- expression analysis tool for identification of biologically relevant associations between phenotypes and gene modules [23]. This approach has been effectively applied in Arabidopsis and rice to detect candidate biomarkers [24]. Uncovering the potential co-regulating gene modules with BGCs would be helpful in exploring the genetic mechanism of how plants detecting allelochemicals, uncover the upstream transcription factors regulating gene clusters and genes or pathways responsible for secreting allelochemicals.

In this study, we conducted a detailed co-expression investigation using time-course transcriptome data generated from mono- and co-cultured rice and barnyardgrass. We identified a candidate BGC in the barnyardgrass genome that is responsible for quercetin glucoside biosynthesis and whose expression is responsive to the presence of rice. We also investigated gene modules and candidate hub genes co-expressed with the functionally well-characterized BGCs in rice (momilactone, phytocassane) and barnyardgrass (DIMBOA, momilactone-like) using co-expression network analysis. Our results provide new insights into the regulatory mechanisms of BGCs responsible for allelopathic interactions between rice and barnyardgrass.

## 2. Results

### 2.1. Transcriptomic Profiling for Allelopathic Interaction between Rice and Barnyardgrass

RNA-Seq was used to investigate the transcriptomic profiles during allelopathic interactions between a rice accession (PI312777) with a high allelopathic potential and barnyardgrass (STB08). For rice, we generated a total of 37.18 GB data, and mapped the clean reads to the rice genome (MSU v7) with an average mapping rate of 87.4% (Table S1). For barnyardgrass, a total of 40.99 GB data were generated, and the average mapping rate was 80.7% (Table S1).

### 2.2. Identification of Candidate Biosynthetic Gene Clusters in Rice and Barnyardgrass

A hybrid pipeline integrating plantiSMASH, metabolic pathway annotation, and co-expression validation was adopted to identify BGCs in the barnyardgrass and rice genomes. A total of 98 BGCs were identified in *E. crus-galli*, while for rice, 46 candidate BGCs were obtained from the PlantiSMASH database. By applying the co-pathway method [11], we reduced the number of BGCs to 11 and 4 in barnyardgrass and rice, respectively (Table S2). We further utilized transcriptomic data to eliminate the BGCs without a gene co-expression pattern, which allowed us to identify seven and two confident candidate BGCs in barnyardgrass and rice, respectively (Table S2).

The two rice BGCs are actually known ones, one is the phytocassane gene cluster in chromosome 2, and another is the momilactone A gene cluster in chromosome 4 [19,25]. Of the seven barnyardgrass BGCs, four were identified in our previous study with a role in biosynthesis of DIBOA-glucoside (PWY-6949) and momilactone A (PWY-7477) [4]. The other three were new with one each involved in biosynthesis of β-caryophyllene (PWY-6275), flavonol (PWY-3101), and quercetin glucoside (PWY-7129) (Table S2).

**Figure 1:**
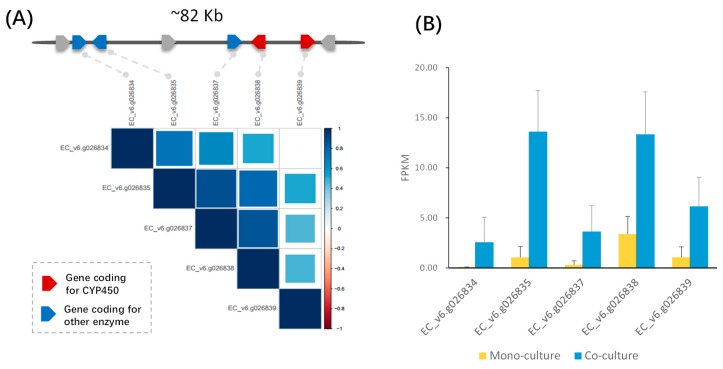
Characterization of the identified candidate quercetin biosynthetic gene cluster. (**A**) Gene co-expression pattern for the genes in the candidate gene cluster. (**B**) Expression changes of the five genes in the quercetin gene cluster in barnyardgrass before and after co-cultured with rice. The expression data are based on our previous RNA-Seq experiment with three biological replicates [4].

Notably, the candidate quercetin glucoside cluster includes two CYP450s, two UDP-glucosyl transferases, and one 2OG-Fe(II) oxygenase, and most of the genes encoding these proteins showed a highly co-expressed pattern (Figure 1A and Table S2). All five genes were significantly up-regulated in barnyardgrass when it was co-cultured (3h) with rice (Figure 1B) based on data previously generated in an experiment with three biological replicates [4]. Quercetin, is a plant flavonol from the flavonoid group of polyphenols, has previously been detected in *E. crus-galli* [26]. Additionally, prior studies established that quercetin and its derivatives are allelochemicals with inhibitory effects on plant growth [27]. Therefore, in addition to the DIMBOA cluster, we found a potential new BGC responsible for quercetin biosynthesis, which could be employed by barnyardgrass against rice during their allelopathic interaction.

### 2.3. Gene Modules Co-regulated with the DIMBOA and Potential Momilactone Gene Clusters in Barnyardgrass

Gene co-expression network was constructed for barnyardgrass based on the transcriptomic profiles. Genes were categorized into 46 co-expression modules, with the module size ranging from 108 to 9413.

**Figure 2:**
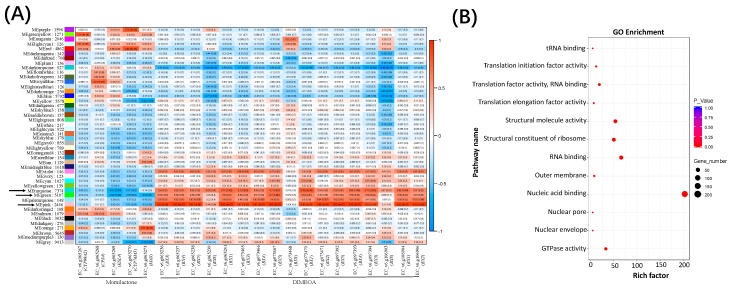
Co-regulated modules and hub genes with biosynthetic gene clusters in barnyardgrass. (**A**) The relationship between the 46 gene modules with genes in the three copies of the 2,4-dihydroxy-7-methoxy-1,4-benzoxazin-3-one (DIMBOA) gene cluster and the presumable momilactone gene clusters in barnyardgrass. Each row corresponds to a module eigengene (correlation between a column and a trait). Each cell contains the corresponding correlation efficient and *p*-value. The arrows indicate three highly co-expressed modules ‘MEpink’, ‘MEturquiise’ and ‘MEgreen’. (**B**) Gene Ontology (GO) enrichment result for the genes in the ‘MEpink’ module.

We then studied the correlations between these 46 modules and the previously identified DIMBOA and candidate momilactone gene clusters in barnyardgrass [4] (Figure 2A). For the presumable momilactone cluster, the ‘MEred’ module was the most significantly and positively (*p* < 0.05, *R^2^* > 0.5) co-expressed with three genes (*CYP99A2*, *KSL4*, and *CYP76M5*) in the cluster. With the same threshold, we found that five modules, namely ‘MEviolet’, ‘MEturquoise’, ‘MEgreen’, ‘MEpaleturquoise’, ‘MEpink’, were positively co-expressed with all three copies of the DIMBOA cluster, but all of them were negatively correlated with the presumable momilactone gene cluster. Among them, the ‘MEpink’ module was the most significantly correlated module (Figure 2A). All five modules had a similar expression pattern and all were significantly and positively correlated with 14 of the 17 genes in the three copies of the DIMBOA cluster, but not with the three copies of *BX8* in the DIMBOA cluster. This observation is consistent with the gene co-expression pattern observed in the maize DIMBOA cluster [28], suggesting that the terminal gene *BX8* in the barnyardgrass DIMBOA cluster might have a unique gene expression signature different from the core genes of the cluster.

Enrichment analyses by AgriGO and MapMan were performed to better understand the possible functional roles of these modules. According to gene ontology (GO) enrichment analysis, the 2446 genes of the ‘MEpink’ module were enriched for 12 GO terms, including ‘GTPase activity’, ‘RNA binding’, ‘translation factor activity’, and ‘nucleic acid binding’ (Figure 2B and Table S3). The ‘MEturquoise’ and ‘MEgreen’ modules were enriched for 28 (7374 genes) and 21 (5107 genes) GO terms, respectively; many were related to mitochondrial, ATP and kinase activity (Table S4). Based on MapMan, 37, 62 and 10 significant categories were significantly associated with the ‘MEpink’, ‘MEturquoise’ and ‘MEgreen’ module, respectively (Table S4). Pathways associated with ‘glucosinolates synthesis’, ‘cytochrome P450′, and ‘phenylpropanoid metabolism’ were also found to be enriched.

In the gene co-expression network analysis, identification of hub genes is vital due to their higher connectivity and consequently more functional activities than the marginal genes. Based on the intramodular connectivity, the top 30 hub genes were selected for each of the three modules. Most hub genes of the ‘MEpink’ module were annotated as cytochrome P450, *O*-methyltransferase, glycine-rich protein family, root cap and terpene synthase (Table S5). Plant *O*-methyltransferases methylate the oxygen atom of various secondary metabolites, and play a key role in lignin biosynthesis, stress tolerance, and disease resistance in plants [29]. Glycine-rich proteins (GRPs) function in plant defense, hormonal signaling, stress responses, and plant cell development [30]. Terpene synthases (TPS) is a big enzyme family in plants, catalyzing the synthesis of diverse terpene molecules [31]. A wide range of plant biological and physiological processes, including environmental adaption, cell growth and development, and defense against biotic and abiotic stressors are controlled by terpenoids [32]. The results of our co-expression analyses suggest that gene clusters for multiple other secondary metabolic pathways together with the DIMBOA gene clusters could be involved in the allelopathic interaction between barnyardgrass and rice.

### 2.4. New Hub Genes Co-regulating with the Two Known Diterpenoid Gene Clusters in Rice

**Figure 3:**
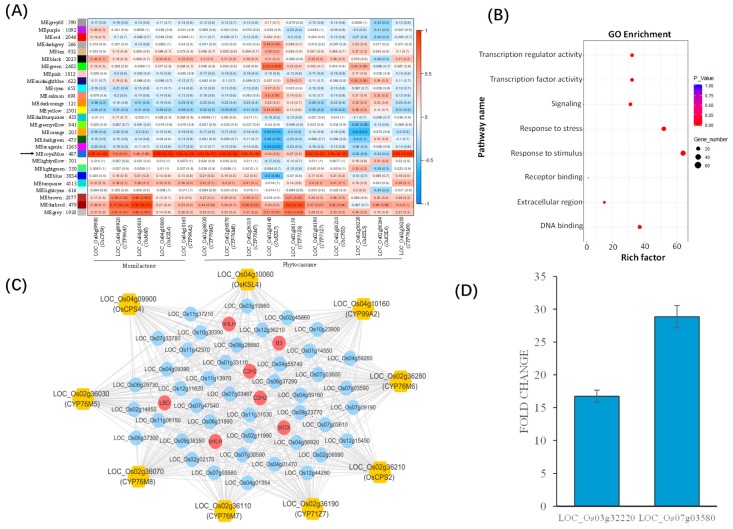
One gene module co-regulated with the two biosynthetic gene clusters in rice. (**A**) The relationship between the 27 gene modules with genes in the momilactone and phytocassane gene cluster in barnyardgrass. Each row corresponds to a module eigengene (correlation between a column and a trait). Each cell contains the corresponding correlation efficient and *p*-value. The arrow indicates the most highly co-expressed ‘MEroyalblue’ module. (**B**) GO enrichment result for the genes in the ‘MEroyalblue’ module. (**C**) The gene co-expression network among the hub genes from the ‘MEroyalblue’ module with genes in the momilactone and phytocassane gene clusters. Transcription factors are colored in red. (**D**) Gene expression changes of the two rice hub genes after co-cultured with barnyardgrass. Expression value of genes without co-cultured with barnyardgrass was normalized to be one. Three biological replicates were used in the experiment.

Based on our expression data of rice, a total of 27 co-expression modules were constructed with the module size ranging from 121 to 4513 members. We then studied the correlations between the two known rice BGCs with the 27 co-expression modules. Interestingly, the ‘MEroyalblue’ module (with 487 genes) was found to be significantly and positively (*p* < 0.05, *R^2^* > 0.5) correlated with the expression of four (*OsCPS4, CYP99A3, OsKSL4, CYP99A2*) of the five genes in the momilactone gene cluster. This module was also co-expressed with six (*CYP76M5, CYP76M8, CYP76M7, CYP71Z7, OsCPS2, CYP76M6*) of ten genes in the phytocassane cluster (Figure 3A).

We further investigated the possible biological roles of the genes in this module. GO enrichment analysis identified eight significantly enriched terms. The most significant ones were in the molecular function category, including ‘transcription regulator activity, ‘transcription factor activity’, ‘DNA binding’, and ‘receptor binding’. In the biological process, GO terms like ‘response to stimulus’, ‘response to stress’, and ‘signaling’ were overrepresented (Figure 3B and Table S6). According to MapMan enrichment analysis, genes involved in regulation of transcription were enriched, particularly transcription factors of the C2H2 and B3 families (Table S6). These observations suggested a possible role of the transcription factors in regulation of the momilactone A and phytocassane gene clusters.

The ‘MEroyalblue’ module had 50 hub genes with seven belonging to five different transcription factor families (B3, bHLH, C2H2, LBD and WOX) (Figure 3C; Table S7). Notably, one of the transcription factors were previous functionally characterized bHLH diterpenoid phytoalexin factor (*DPF*, *LOC_Os01g00990*), which has been confirmed to regulate the expression of genes in both the momilactone and phytocassane clusters [21]. In addition, we also identified a cluster of five genes encoding ‘SCP-like extracellular protein’ with the PFAM ‘Cysteine-rich secretory protein family’ encoding gene in the hub gene list, suggesting a possible role of the module in secreting allelochemicals. Based on qRT-PCR, both the C2H2 family transcription factor, *LOC_Os03g32220*, and the gene (*LOC_Os07g03580*) encoding cysteine-rich secretory protein were significantly upregulated in barnyardgrass co-cultured with rice (Figure 3D), supporting a role of the genes in allelopathic interaction between rice and barnyardgrass.

### 2.5. Putative Upstream Genes of the DIMBOA and Presumable Momilactone Biosynthetic Gene Clusters in Barnyardgrass

**Figure 4:**
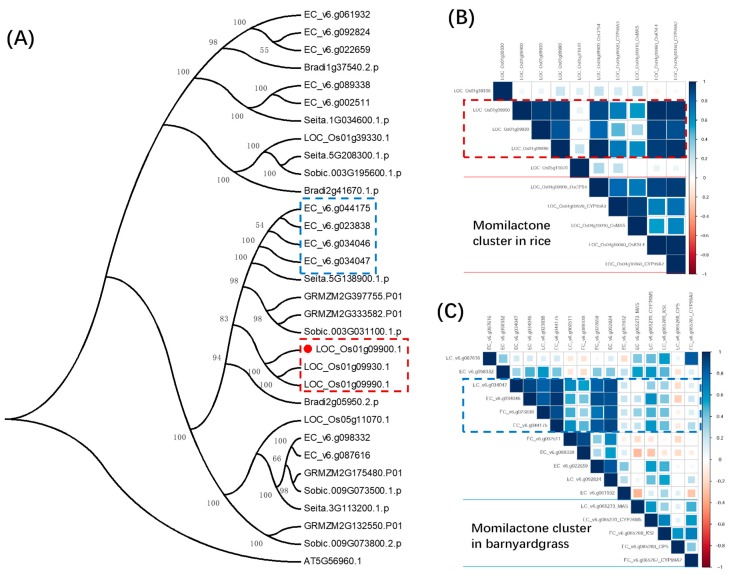
Co-expression pattern between genes in the momilactone cluster and transcription factor DPFs. (**A**) Phylogenetic tree for rice diterpenoid phytoalexin factor (DPF) and its homologous genes. The rice *DPF* (*LOC_Os01g09900*) gene is highlighted with red dot, while its paralogs is highlighted with red dashed frame. The orthologous DPFs in *E. crus-galli* are highlighted in the blue dot box. (**B**) The co-expression coefficient matrix for genes in the momilactone cluster and DPFs in rice. The rice *DPF* genes regulating the rice BGCs are highlighted with red dot box. (**C**) The co-expression coefficient matrix for genes in the predicted momilactone cluster and DPF homologs in barnyardgrass.

It has been previously demonstrated that DPF (*LOC_Os01g09900*) acts as a master transcriptional regulatory factor for diterpenoid phytoalexin biosynthesis in rice [33]. Given that the momilactone cluster is currently only found in rice and barnyardgrass, it was of interest to know the potential roles of the barnyardgrass genes orthologous to the rice DPF. Based on the phylogenetic analysis, we found four DPF orthologs in barnyardgrass (Figure 4A). When examining the co- expression pattern of these barnyardgrass *DPF* orthologs with the genes in the momilactone gene cluster in rice and barnyardgrass, we observed a different pattern. In rice, three genes (1 *DPF* and 2 *DPF-like*) were highly co-expressed with the genes of the momilactone cluster, particularly with *CYP99A2*, *OsKSL4*, and *OsCPS4* (Figure 4B). In barnyardgrass, however, we did not observe a highly co-regulated relationship between the *DPF* orthologs and genes in the presumable momilactone gene clusters (Figure 4C), suggesting that the regulatory mechanism for momilactone biosynthesis might be different in the two species.

**Figure 5:**
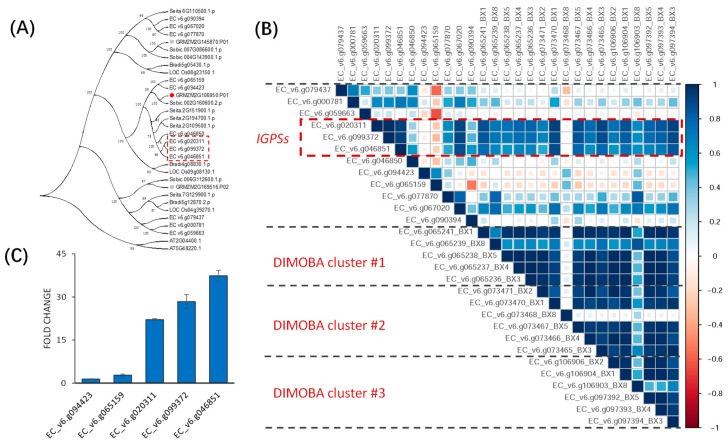
Co-expression pattern between genes in three copies of the DIMBOA cluster and *IGPS* orthologs in barnyardgrass. (**A**) Phylogenetic tree for genes homologous to *IGPS* across 6 grass species and *Arabidopsis thaliana*. The gene encoding indole-3-glycerolphosphate synthase (*IGPS*) in maize is highlighted in the red dotand the two homologous genes in maize are indicated by grey dots. Barnyardgrass orthologs are boxed in red dot box. (**B**) The co-expression coefficient matrix for genes in the three copies of the DIMBOA cluster and *IGPS*s in barnyardgrass. (**C**) Gene expression changes of the 5 barnyardgrass *IGPS*s after co-culturing with rice. Expression value of genes without co- culturing with rice was normalized to be one. Three biological replicates were used in the experiment.

The first step in benzoxazinoid biosynthesis is catalyzed by indole-3-glycerolphosphatelyase (*BX1*), which converts indole-3-glycerolphosphate into indole. The maize genome contains three genes encoding indole-3-glycerol phosphate synthase (*IGPS*) (with Pfam ‘PF00218′). A previous study found that only one (*GRMZM2G106950*) of the three *IGPS* genes was highly co-expressed with the core BX genes (*BX1-BX5*) in the benzoxazinoid pathway, and this gene was suggested to catalyze the reaction directly upstream of *BX1* [28]. The *E. crus-galli* genome contains 12 putative indole- 3- glycerol phosphate synthases (with Pfam ‘PF00218). Phylogenetic analysis suggested that six of the 12 genes are orthologs of the maize *IGPS* (Figure 5A), and three of the six were highly co- expressed with the core *BX* genes (*BX1-BX5*) in all three copies of the DIMBOA cluster (Figure 5B). qRT-PCR analysis showed that the three *IGPS* genes are significantly over-expressed in barnyardgrass co-cultured with rice than when grown alone (Figure 5C). These results suggest that, just like in maize, these three barnyardgrass *IGPS* genes are likely regulators of the genes of the DIMBOA clusters, and that they could function in the allelopathic interaction between barnyardgrass and rice.

## 3. Discussion

The discovery of new plant BGCs has been facilitated by the increasing availability of assembled plant genomes and the development of novel bioinformatics tools for gene cluster discovery. Using the PlantiSMASH software, we identified a total of 46 and 98 candidate gene clusters in the rice and barnyardgrass genome, respectively. However, the candidate gene clusters were narrowed down to two (rice) and seven (barnyardgrass) when we applied additional methods like co-pathway and co- expression analyses. A previous study found that many bioinformatically predicted BGCs in plants are typically not co-expressed, and thus many of them may not be true BGCs that correspond with actual secondary metabolic pathways [28]. Our results presented here together with results from prior studies suggest that it is essential to use multiple approaches in predicting BGCs with a potential biological role. The final candidate gene clusters identified in this study meet the criteria of physical proximity, co-expression, and within the same pathway, and thus should be relatively reliable. However, further experimental validation is needed to verify their BGC identity.

Interestingly, one of the identified BGCs is annotated to be involved in quercetin biosynthesis. In addition to the potential allelopathic effect on plants [27], quercetin is claimed to be a versatile antioxidant with protective abilities against tissue injury induced by various drug toxicities [34]. Quercetin and its derivatives have been detected in *E. crus-galli*, and reported to have significant activities against the human carcinoma [26], consistent with traditional use of *E. crus-galli* as medication. Therefore, this predicted quercetin cluster could also have the potential to be used in development of medicines via metabolic engineering approaches.

Co-expression network analysis can be used for investigation of thousands of genes with identical expression patterns, clustering, and instantaneous recognition over various situations [35]. It is an important and magnificent method for gene interaction analysis and interpretation of molecular mechanism in numerous species [35]. When performing the co-expression network analysis, we observed that the genes in the rice momilactone and phytocassane BGCs were positively co-expressed and had a common co-regulated module ‘MERoyalblue’ (Figure 3). This is likely due to the fact that both clusters produce the same secondary compounds, diterpenoid phytoalexins, derived from a common precursor, geranylgeranyl diphosphate (*GGDP*) [21]. In addition, it has also been reported that the *DPF* transcription factor can activate not only *OsCPS2* expression for phytocassane biosynthesis, but *CYP99A2* for momilactone biosynthesis. However, a different expression pattern was observed in barnyardgrass. Here, the expression of genes in the DIMBOA cluster and presumable momilactone clusters were negatively co-expressed (Figure 2). Based on our previous findings [4], when barnyardgrass is co-cultured with rice, the genes within all three copies of the DIMBOA clusters were significantly upregulated. Therefore, we propose that, unlike the momilactone cluster in rice, the function of the likely momilactone cluster in barnyardgrass has changed from allelopathic interaction to another. This is supported by the different co-expression pattern of the orthologous bHLHs in the two species.

The discovery of the involvement of biosynthetic gene clusters for allelopathic interactions between rice and barnyardgrass has brought about more implications for the molecular mechanisms of crop–weed interaction. Herein we explored and discovered genetic modules and candidate hub regulatory genes via co-expression and pathway analysis. In future, a couple of interesting issues remain to be resolved. For example, chromatin analyses by Hi-C or ChIP-seq could be performed to better understand the regulatory mechanism involved in the biosynthetic pathways. How did the barnyardgrass evolve to acquire the DIMBOA and candidate momilactone gene clusters which are present in the genomes of two most staple crops (rice and maize) of the world? In addition, for rice breeding, bulked segregant analysis and genome wide association methods could be applied for investigating the adaptive alleles or genes which confer resistance to DIMBOA.

## 4. Materials and Methods

### 4.1. Plant Materials and Growth Conditions

Barnyardgrass (STB08) seeds picked up from rice paddy fields in the lower Yangtze River area of China (30°17′ N, 119°57′ E) and rice (PI312777) seeds were collected from Chui-Hua Kong’s laboratory at China Agricultural University, Beijing, China. The rice–barnyardgrass allelopathic interaction experiment was performed as previously described [4]. The RSA (relay seeding in agar) method with minor modifications [36] was used to investigate the allelopathic interactions between barnyardgrass (STB08) and rice (PI312777). Ten germinated rice seeds (PI312777) were first transferred to a bottle (10 cm in base diameter) with 50 mL of 0.5% agar medium, and germinated barnyardgrass seeds (STB08) were transferred to a Petri dish with sterile water. The PI312777 seeds were ordered in three rows with a 3-4-3 pattern. Ten germinated STB08 seeds were then transferred between two rows in the same bottle containing the rice seeds. The PI312777 and STB08 seedlings were co-cultured for 3 h, 3 d, 7 d, and 14 d in a SAFE incubator (Ningbo, China) with a day/night regime of 14 h (30 °C) and 10 h (20 °C), and 75% relative humidity. Mono-cultured PI312777 and STB08 grown in the same conditions were used as controls. For the RNA-Seq experiment, ten mono- and co-cultured PI312777 and STB08 plants were collected from each time point, instantly frozen in liquid nitrogen, and kept at –80 °C until use. A total of 32 RNA-seq datasets were generated, 16 each for rice and barnyardgrass (Table S1).

### 4.2. Analysis of RNA-seq Data

Illumina RNA-Seq libraries from PI312777 and STB08 were prepared and sequenced on a HiSeq 2000 system following the manufacturer’s instructions. Data were filtered before the downstream analysis to decrease data noise. NGSQC Toolkit version v2.3.3 was applied in quality control and filtering with the default settings [37]. TopHat2 [38] was used to map the reads to the rice reference genome (MSU7.0, http://rice.plantbiology.msu.edu/) and *E. crus-galli* reference genome (http://ibi.zju.edu.cn/RiceWeedomes/Echinochloa/) [4]. After alignment, the Cufflinks package was used to measure the FPKM (fragments per kilobase per million reads) value of each gene.

### 4.3. Metabolic Pathway Annotation

To build the *E. crus-galli* metabolic enzyme database, The Ensemble Enzyme Prediction Pipeline (E2P2 v3.0, https://dpb.carnegiescience.edu/labs/rhee-lab/software) was used for enzymatic annotations of the *E. crus-galli* coding genes [11]. Using the enzymatic annotations, PathoLogic [39] version 21.0 implemented in Pathway Tools was then applied to construct the metabolic pathway database [40]. The metabolic pathway annotation file of *O. sativa* was downloaded from Plant Metabolic Network v13.0 (https://www.plantcyc.org/).

### 4.4. Prediction of Candidate Biosynthetic Gene Clusters by PlantiSMASH

The online computational tool kit PlantiSMASH [10] was utilized to predict candidate biosynthetic gene clusters in the barnyardgrass genome. The rice gene clusters predicted by this software were fetched from http://plantismash.secondarymetabolites.org/precalc/results/Oryza_sativa_Japonica/.

### 4.5. Gene Cluster Validation by Co-pathway and Co-expression Analyses

The candidate gene clusters predicted by plantiSMASH were first annotated with the species- specific metabolic pathway database and further filtered with the following approaches. (1) Co-pathway: we used two criteria to define co-pathway metabolic gene clusters, (a) at least two genes annotated with the same pathway ID (MetaCyc pathway identifier), (b) genes of the same cluster classified into at least two different reactions (MetaCyc reaction identifier); (2) co-expression: first, Pearson correlation coefficient (PCC) was calculated for all the gene pairs within a characterized metabolic gene cluster, and the number of pairs showing significant co-expression (*p*-value < 0.05) was calculated; second, PCC was calculated for all the metabolic gene pairs, and its distribution was estimated [11], we then took the 95th percentile as the PCC threshold to select co-expressed genes. Co-expression coefficient matrix was visualized using the R package ‘corrplot’ [41].

### 4.6. Co-expression Network Investigation

WGCNA was used to build the gene co-expression networks [23]. All genes, except those not expressed in all time points, were used in clustering, and the gene dendrogram was employed in module identification by the dynamic tree cut method (mergeCutHeight = 0.25, maxBlockSize = 8000) for both rice and barnyardgrass. The *β*, a crucial parameter for network building, was chosen as suggested in the user’s guide to adjust both the scale-free topology and appropriate node connection. Then, the modules were examined for their connections with the trait by correlating module eigengenes (MEs) with trait measurements. BGCs of rice (momilactone, phytocassane) and barnyardgrass (DIMBOA, momilactone) were considered traits. Module eigengenes were regarded as the critical components in the principal component analysis for the individual gene module, and the expression arrangement of all genes could be outlined into a single typical expression profile within an assumed module. Furthermore, significant modules were selected based on *p*-value < 0.05.

### 4.7. Module Hub Gene Analysis and Visualization

Hub genes, the utmost central and linked genes, engage in many interactions and play a more significant function than other genes of the network [42]. The MM (module membership) was specified as the correlation of gene expression profile with ME, and the GS (gene significance) measure was defined as (the absolute value of) the correlation between gene and traits. We took the absolute value in both MM and GS measurement. In WGCNA, the connectivity level was defined as the sum of all weight through all edges of a node. We used MM > 0.8, GS > 0.2 and the highest intramodular connectivity in identification of the hub genes for both barnyardgrass and rice. The co- expression interactions and patterns of the top hub genes of each barnyardgrass module and all significant rice hub genes were visualized using Cytoscape v 3.6.1 [43].

### 4.8. Enrichment Analyses of Gene Modules

AgriGO with ‘*Oryza sativa* MSU7.0’ set as the species background (http://bioinfo.cau.edu.cn/agriGO/) was used for the GO enrichment analysis. GO enrichment terms were selected based on *p*-value (0.0001) and the false discovery rate (0.05). Furthermore, we used MapMan in investigating the complex metabolic pathways and biological process, and the PlantTFDB 4.0 database (http://planttfdb.cbi.pku.edu.cn/prediction.php) in analyzing rice TFs [44].

### 4.9. Orthologous Gene Identification

To identify the *E. crus-galli* genes orthologous to genes encoding rice bHLH (LOC_Os01g09990) and maize indole-3-glycerolphosphate synthase (GRMZM2G106950), we created a protein database by combining protein sequences of six grass species (including *O. sativa*, *Z. mays*, *B. distachyon*, *S. bicolor*, *S. italica*, and *E. crus-galli*) and *A. thaliana* (downloaded from Phytozome v9.0 except *E. crus- galli*). BLASTP was then used to scan homologous genes encoding the two genes in the protein database (E-value thresholds: 10^−10^). RAxML v8 was applied with the parameters ‘- m PROTGAMMAAUTO -auto-prot = bic’ to automatically select the best protein model for tree construction [45]. Each tree was constructed with 100 bootstraps. MEGA v5.1 [46] was applied to draw the constructed tree.

### 4.10. Gene Expression Validation Using Quantitative Real-Time PCR (qRT-PCR)

RNA was extracted utilizing the Trizol (Invitrogen, Carlsbad, CA, USA) method following the manufacturer’s protocol. The PrimeScript™ RT Reagent Kit with gDNA Eraser and SYBR Premix ExTaq™ (Takara, Japan) was used in cDNA generation and qRT-PCR based on the manufacturer’s instruction. qRT- PCR was carried out using the LightCycler^®^ 96 System (Roche, Indianapolis, IN, USA). The qRT-PCR was setup following the protocol described previously [47]. Three biological replicates were used, and the values shown are the mean ± SD, where the statistical significance evaluated using the Student’s *t*-test. The rice and barnyardgrass actin genes were used as internal controls for normalizing the variance in rice and barnyardgrass, respectively. The primers used in qRT-PCR are shown in Table S8.

## 5. Conclusions

To achieve sustainable agriculture with less chemical usage, it is desirable to better understand the genetic mechanism underlying crop–weed interactions and develop competitive crop cultivars. Allelopathy through secondary metabolites is one of the central processes underlying crop–weed interactions, and biosynthetic gene clusters have been proved to convey genomic mechanism governing these allelopathic interactions. In this study, we not only identified candidate new biosynthetic gene clusters but also applied gene correlation network to identify gene modules and hub genes associated with BGCs involved in the allelopathic interactions. This study provides fresh insights into the secondary metabolic pathways and related regulatory mechanism involved in the rice–barnyardgrass allelopathic interaction.

## Supplementary Materials

The following are available online at https://www.mdpi.com/1422-0067/20/16/3846/s1. Table S1: Summary of the RNA-seq data for rice and baryardgrass in this study. Table S2: Predicted candidate biosynthetic gene clusters in the rice and barnyardgrass genomes. Table S3: GO enrichment for genes in the gene modules associated with the expression of the DIMBOA and momilactone gene clusters in barnyardgrass. Table S4: MapMan functional enrichment for genes in the gene modules associated with the expression of the DIMBOA and momilactone gene clusters in barnyardgrass. Table S5: Top 30 hub genes for each module associated with the DIMBOA and momilactone cluster in barnyardgrass. Table S6: Functional enrichment for the rice MEroyalblue module. Table S7: Hub TFs in the modules associated with the momilactone and phytocassane cluster in rice. Table S8: Primers used in qRT-PCR in this study.

## Abbreviations

BGCs	Biosynthetic gene clusters
DPF	Diterpenoid phytoalexin factor
WGCNA	Weighted gene co-expression network analysis
*BX1*	Indole-3-glycerolphosphatelyase
IGPS	Indole-3-glycerolphosphate synthase
PCC	Pearson correlation coefficient
MM	Module membership
GS	Gene significance
qRT-PCR	quantitative real-time PCR
